# Assessment of health education products aimed at controlling and preventing helminthiases in China

**DOI:** 10.1186/s40249-019-0531-y

**Published:** 2019-03-26

**Authors:** Men-Bao Qian, Chang-Hai Zhou, Hui-Hui Zhu, Ting-Jun Zhu, Ji-Lei Huang, Ying-Dan Chen, Xiao-Nong Zhou

**Affiliations:** 1 National Institute of Parasitic Diseases, Chinese Center for Disease Control and Prevention, Shanghai, 200025 China; 2 Key Laboratory of Parasite and Vector Biology, Ministry of Health, Shanghai, 200025 China; 3 National Center for International Research on Tropical Diseases, Shanghai, 200025 China; 4 World Health Organization Collaborating Center for Tropical Diseases, Shanghai, 200025 China

**Keywords:** Health education, Health education product, Helminthiasis, Evaluation, China

## Abstract

**Background:**

Helminthiases have placed a huge burden of disease on the population in China. However, widespread control activities have led to significant achievements. As health education has been widely disseminated and plays an important role in the control and elimination of these diseases, we collected health education products aimed at controlling and preventing helminthiases in China. We analyzed their characteristics and assessed their quality.

**Methods:**

Firstly, health education products aimed at controlling and preventing helminthiases were collected from a diverse range of organizations. Secondly, the expert brainstorming and Delphi methods were applied to establish an evaluation system, which was then used to assess the collected products systematically. Those deemed excellent were awarded. Characteristics – including type, source, targeted disease(s), targeted population, and languages – of the collected products and the awarded products were presented here.

**Results:**

In total, 96 health education products on helminthiases were collected from 53 organizations. Most products belonged to either the graphic design (47) or daily-use (24) category. Seventy were collected from Centers for Disease Control and Prevention and 20 from institutes or control stations of parasitic diseases, primarily at the provincial and county levels. Regarding disease targets of the products, 67 focused on a single helminthiasis, 25 on multiple helminthiases, and the remaining four on non-specific diseases. Of the 67 single helminthiasis-focused products, most targeted schistosomiasis (37), followed by echinococcosis (16). The majority of products (79) targeted the general population, while 11 targeted students specifically. Regarding languages, 86 products were only in Chinese, while the other ten were in both Chinese and the minority languages of China. Out of these ten products, one targeted schistosomiasis and the other nine targeted echinococcosis. Thirty-four products were awarded. The characteristics of the awarded products were similar to those of the collected products.

**Conclusions:**

A diverse range of health education products have been designed and applied for the prevention and control of helminthiases in China. Many products have good features such as specifying the targeted diseases and populations. However, there are significant gaps in terms of both the quantity and quality of products pertaining to some of the diseases. Experiences from the awarded products could be drawn upon to design more products aimed at a range of different helminthiases.

**Electronic supplementary material:**

The online version of this article (10.1186/s40249-019-0531-y) contains supplementary material, which is available to authorized users.

## Multilingual abstracts

Please see Additional file 1 for translation of the abstract into the five official working languages of the United Nations.

## Background

Helminthiases have historically placed a high disease burden on the population in China, especially in less developed areas [1]. Most helminthiases are under control due to widespread control efforts, but some still present an important public health problem [1].

Schistosomiasis has ranked as the top public health priority in China since the 1950s. It was estimated that about 11.6 million people were infected in the 1950s, reducing to about 54 000 in 2016 [2, 3]. Recently another zoonotic helminthiasis, echinococcosis, has received great attention from the central government due to its high disease burden in western China [4, 5]. It is estimated that about 166 000 people are currently infected [6].

In China, the prevalence of soil-transmitted helminthiases reached 53.6% in the early 1990s, and decreased to 19.6% in the early 2000s based on national surveys [7]. The prevalence further decreased to 3.12% in 2013 based on surveillance data, however, some highly endemic areas still exist in western China [8]. Diverse food-borne helminthiases are likewise endemic in China due to various raw-eating habits (e.g. fish, meat, and vegetables). Of the 15 million people across the globe who have clonorchiasis, 13 million are distributed in China, especially in the eastern areas [9, 10]. Outbreaks of paragonimiasis have been reported in different areas [11]. Additionally, other important food-borne helminthiases including fascioliasis, sparganosis, and angiostrongyliasis are locally endemic [1, 12–14].

Diverse control measures are usually adopted to control these helminthiases, with health education playing an important role due to its ability to increase knowledge, modify attitudes, and change behaviors among the population [15–17]. Thus, health education is usually integrated into control strategies against helminthiases [18, 19]. For example, chemotherapy, the main method used in the control of many helminthiases, could not alone prevent reinfection without patients also modifying their personal hygiene [20, 21]. The integration of health education can promote a change in behavior, which ensures the sustainability of chemotherapy [22]. Furthermore, health education can help the achievement of other measures (e.g. compliance in chemotherapy) [23]. Health education is influenced by many factors including the quality of the health education products, the delivery platform, the characteristics of targeted communities, as well as human behavior [17]. To determine the characteristics and assess the quality of health education products aimed at controlling and preventing helminthiases in China, a national analysis and evaluation study was carried out in 2016.

## Methods

### Collection of health education products

Diverse approaches were applied to increase the coverage of the collection of health education products. First, a call-out for health education products was announced through the National Office Automatic System for Disease Control, which covers the Centers for Disease Control and Prevention (CDCs) and institutes or control stations of parasitic diseases (IPDs) in China. Second, it was further announced on the website of the National Institute of Parasitic Diseases, China CDC, which is the national technical organization for the research and control of parasitic diseases. Third, due to the increasing influence of digital media, it was also announced through two WeChat public accounts, namely “Health in People’s Health” and “Healthy China”.

Products were collected from April to May 2016. A short description from submitted organization was attached for each product to introduce the content. All collected products were sorted and numbered. Characteristics – including type, source, targeted disease(s), targeted population, and languages – were extracted for each product.

### Evaluation of health education products

A core expert panel, including 10 national experts on health education and/or helminthiases control, was first established. The core expert panel convened and defined the classification of the products and formulated the evaluation indicators for different types of products. Products were classified into four types: graphic design products, daily-use products, audiovisual products, and comprehensive products. Comprehensive products were defined as those providing a combination of the other three types. Four primary indicators and 12 secondary indicators were included (see Additional file 2: Tables S1–S3).

Second, two rounds of Delphi survey were implemented to capture the weight of each indicator. This enrolled 23 experts including the core expert panel. The procedures and results of the Delphi survey are described in detail elsewhere [24]. In brief, each expert ranked the importance of each indicator with a value ranging from 5 (highest importance) to 1 (least importance). The experts also provided evidence on how they judged. The evidence belonged to one of four groups, namely theoretical analysis, practical experience, experience from peers, and intuition. Each evidence group was valued using a three-level scale (high, moderate, and low). Meanwhile, the experts’ familiarity with each indicator was captured, also ranging from 5 (highest familiarity) to 1 (least familiarity). Based on the importance, evidence of judging, and familiarity, an overall value was calculated from each expert for each indicator. The weight of each indicator was calculated based on the average of all experts (Additional file 2: Tables S1–S3).

Third, the core expert panel convened again to assess the products. Eight experts participated, while the other two could not attend. First, each expert selected several of the best products of each type from all collected products (Additional file 2: Table S4). Staff members from National Institute of Parasitic Diseases, China CDC summarized the results. Those that made it through the initial screening went to the next round. Then, each expert scored the secondary indicators for each product selected. To increase equity, the lowest and highest scores of each secondary indicator were excluded. Then, the average score was calculated for each indicator and multiplied by the corresponding weight from the Delphi survey. The total score of a product was calculated with the addition of the scores of the 12 indicators. Based on the total score, a product was classified into a class (first, second, third, fourth, and others). Classes 1–4 were awarded.

## Results

### Characteristics of the collected products

#### Types of products

A total of 96 health education products on helminthiases were collected from 53 organizations. Among these, 47 were graphic design products, 24 were daily-use products, 11 were audiovisual products, and 14 were comprehensive products. Of the graphic design products, there were 17 display boards, 11 posters, nine foldout brochures, and seven handbooks. Of the daily-use products, most were household objects (17), while the other seven comprised learning products and puzzles. Of the audiovisual products, nine were videos and two were WeChat products. Of the comprehensive products, six were a combination of graphic design and daily-use products and four were a combination of graphic design, daily-use, and audiovisual products (see Table 1).

**Table 1:** Characteristics of collected and awarded health education products aimed at controlling and preventing helminthiases in China (types and sources)

Classification	Sub-classification	Number of collected products	Number of awarded products
Type			
Graphic design products		47	9
	Display board	17	1
	Poster	11	2
	Foldout brochure	9	2
	Handbook	7	2
	Comic strip	1	1
	Leaflet	1	1
	Pictorial	1	0
Daily-use products		24	9
	Household^a^	17	8
	Learning^b^	4	0
	Puzzles^c^	3	1
Audiovisual products		11	8
	Video	9	6
	WeChat	2	2
Comprehensive products		14	8
	Graphic design and daily-use	6	4
	Graphic design, daily-use, and audiovisual	4	3
	Other	4	1
Source			
CDCs		70	21
	National level	2	1
	Provincial level	38	12
	Prefectural level	6	2
	County level	24	6
IPDs		20	11
	Provincial level	14	7
	County level	6	4
University		2	0
Private company		1	0
Hospital		1	0
Non-governmental organization		1	1
Overseas organization		1	1

*CDCs* Centers for disease control and prevention, *IPDs* Institutes or control stations of parasitic disease

^a^Including condiment dispenser, fan, desk calendar, umbrella, table cloth, towel, apron, bag, flashlight, etc.

^b^Including pencil case, mouse pad

^c^Including magic cube, coloring card, jigsaw puzzle

#### Sources of products

Of the 96 products, 70 were collected from CDCs and 20 from IPDs. Two products were collected from a university, while only one each was collected from a hospital, private company, non-governmental organization, and overseas organization.

Of the 70 products from the CDCs, two were collected at the national level, 38 at the provincial level, six at the prefectural level, and 24 at the county level. Of the 20 from the IPDs, 14 came from the provincial level and six from the county level (see Table 1).

#### Targeted diseases of products

Helminthiases in this study were classified into three groups, namely soil-transmitted, food-borne, and zoonotic helminthiases.

Of the 96 products, 67 focused on single helminthiasis, 25 on multiple helminthiases, and the other four on non-specific diseases.

Of the 67 products targeting single helminthiasis, two targeted soil-transmitted helminthiases (enterobiasis), 12 targeted food-borne helminthiases, and 53 targeted zoonotic helminthiases. Of the 12 products targeting food-borne helminthiases, five focused on clonorchiasis, four on paragonimiasis, and three on sparganosis. Of the 53 products targeting zoonotic helminthiases, 37 focused on schistosomiasis and 16 on echinococcosis.

Of the 25 products targeting multiple helminthiases, six focused on soil-transmitted helminthiases, five on food-borne helminthiases, and 11 on soil-transmitted helminthiases and food-borne helminthiases (see Table 2).

**Table 2:** Characteristics of collected and awarded health education products aimed at controlling and preventing helminthiases in China (targeted diseases, targeted population, and languages)

Classification	Sub-classification	Number of collected products	Number of awarded products
Targeted disease			
Single diseases		67	22
Soil-transmitted helminthiases		2	0
	Enterobiasis	2	0
Food-borne helminthiases		12	3
	Clonorchiasis	5	0
	Paragonimiasis	4	2
	Sparganosis	3	1
Zoonotic helminthiases		53	19
	Schistosomiasis	37	13
	Echinococcosis	16	6
Multiple diseases		25	11
Soil-transmitted helminthiases		6	2
Food-borne helminthiases		5	3
Soil-transmitted and food-borne helminthiases		11	5
Other		3	1
Non-specific		4	1
Targeted population			
General population		79	27
Students		11	3
Fishermen and boatmen		2	2
Internet users		2	2
Decision-makers		1	0
People visiting doctors		1	0
Language			
Chinese		86	28
Combination of Chinese and minority languages		10	6
	Schistosomiasis	1	1
	Echinococcosis	9	5

#### Targeted population of products

Most products (79 products) targeted a non-specific population, namely the general population. Eleven products targeted students. Two products relating to health education on schistosomiasis targeted fishermen and boatmen. Another two products targeted internet users through WeChat (see Table 2).

#### Languages of products

Most (86) products were in Chinese, while the other 10 were in both Chinese and other minority languages of China. One product in multiple languages targeted schistosomiasis and the other nine targeted echinococcosis (see Table 2).

### Characteristics of the awarded products

In total, 34 products were awarded. The characteristics of the awarded products were similar to those of the collected products. Most came from the CDCs (21) and IPDs (11) at the provincial and county levels (see Table 1).

Most awarded products focused on schistosomiasis (13) and echinococcosis (6). Most (27) awarded products targeted the general population. Of 11 collected products targeting students, three were awarded. Although there were not many collected products for fishermen and boatmen (2) and internet users (2), all of these products were awarded. Although the proportion of the products in multiple languages was only 10.4% (10/96), the proportion of multiple-language awarded products reached 17.6% (6/34) (see Table 2).

#### First-class products

From each type of product, one was selected as first class. The first-class graphic design product was a comic strip aimed at schistosomiasis prevention targeting students, submitted by a provincial CDC. Through the combination of images and words, this product imparts a lot of knowledge on schistosomiasis and how to prevent it. Owing to the vivid images, it is suitable for students in endemic areas.

The first class daily-use product was a condiment dispenser for the control of food-borne helminthiases targeted at adults, submitted by a provincial IPD. A slogan to encourage the prevention of parasitic diseases is printed at the top of the condiment dispenser. Along the side, three best practices are shown, namely the washing of fruits and vegetables, the separate preparation of cooked and uncooked foods, and the thorough cooking of meat. As this dispenser is meant for daily use, the messages can be seen frequently and thus increase the prevention of food-borne helminthiases.

A video named *Rhyme of Poyang Lake* was awarded first class in the audiovisual category. It was submitted by a provincial IPD and targeted the general population. The video shows a boy going to his grandma’s home, touching contaminated water, and then becoming infected with *Schistosoma*. The story imparts basic knowledge of schistosomiasis and how to prevent to the viewer. This video could be screened not only in school for students, but also for their guardians.

Regarding the comprehensive products, first class was awarded to a group of products aimed at the prevention of echinococcosis. It integrates many types of products, including graphic design, daily-use, and audiovisual, which all target a diverse population, thereby potentially increasing the coverage of the targeted population. These products are both in Chinese and Tibetan. For example, a wall calendar highlights the importance of echinococcosis prevention. A teapot is printed with information on how to prevent echinococcosis. As the teapot is an important daily-use product for Tibetans, the information is likely to reach each family member on a daily basis. A music video introducing echinococcosis prevention through a pleasant song is another component.

## Discussion

This is the first national assessment and analysis of health education products aimed at controlling and preventing helminthiases in China. The survey found that diverse health education products have been designed for the control of helminthiases in China, with several important features that are expected to benefit the population’s understanding and promote the application of health education in the control of helminthiases in China.

Most of the collected products (74%) belong to the graphic design category and daily-use category. This is understandable as the design and production of these products, especially the former category, are simpler and cheaper, and they are more easily distributed, resulting in wider coverage. Both categories include diverse types of products. Display boards and posters dominated the graphic design category. The advantage of these is that they can disseminate the knowledge in public, while other products such as the foldout brochure and handbook must be distributed to each person. Most daily-use products were household items, as these are easily accessible and can thus target a larger portion of the population. Although videos are complex and expensive to make, 13 videos (nine in the audiovisual category and four in the comprehensive category) were collected. Demonstrations through video are more interesting and engaging, and knowledge can be shown vividly, namely in an intuitionistic approach [15]. In addition, there are now more opportunities for videos to be broadcast due to China’s social and economic development. For example, many schools in both urban and rural areas are now equipped with multimedia [25]. Recently a social media platform, WeChat, has become highly popular, with daily users reaching 570 million in 2016, thus making it an attractive option for health education [26]. As such, two WeChat products on helminthiases were collected. However, it should be emphasized that whilst WeChat creates the potential for high health education coverage, it is the quality of these products that will determine whether many internet users will read them. It should also be noted that some people in remote areas endemic with helminthiases are not accessible through this type of product. Comprehensive products have an advantage over single-type products, namely that they can cover a larger portion of the population.

Most products were submitted by the CDCs and IPDs, which is consistent with the fact that these bodies are predominantly in charge of controlling helminthiases, especially when it comes to widespread interventions. Because interventions are implemented by these organizations at the provincial and county levels, most products were designed and distributed by the CDCs and IPDs at these levels, especially at the provincial level where there is higher capacity [27]. Only six products were collected from other organizations, namely a university, hospital, private company, non-governmental organization, and overseas organization. This demonstrates the low participation of these organizations in the education on helminthiases in China.

Most products targeted a single disease and thus had high pertinence. The collected products predominantly targeted schistosomiasis and echinococcosis. As schistosomiasis has been a high priority of public health in China since 1950s [28], health education has been integrated into the national control strategy against schistosomiasis [17, 29]. Special institutes or control stations for schistosomiasis have been established in many schistosomiasis-endemic areas in China [27]. Recently, echinococcosis has received a lot of attention from the central government and thus health education aimed at this disease has also been given high priority [30]. Huge resources are thrown into the control of echinococcosis, promoting the design and distribution of health education products. Additionally, many products also targeted multiple diseases. This makes sense as some helminthiases have similar transmission routes, and the integration of diverse helminthiases in the same product is an economical approach.

Most products targeted the general population, with a significant portion targeting students. To target the general population is economical, while to target students is highly pertinent. Echinococcosis is endemic in western China, where many minority groups live [4, 6], and thus many products on echinococcosis are in multiple languages (e.g. Chinese and Tibetan).

Similar to the characteristics of collected products, the awarded products were also mainly collected from the provincial- and county-level CDCs or IPDs. Three first-class products targeted schistosomiasis and echinococcosis, which is understandable considering the high output of products on these diseases. These first-class products were of high quality and can be drawn upon to design other products in the future. The comic strip for schistosomiasis prevention (first class in the graphic design category) had abundant knowledge, which guides how to prevent schistosomiasis. The design and layout are fine, which could appeal the attention from students. The condiment dispenser for the control of food-borne helminthiases (first class in the daily-use category) is very practical. It can be seen when preparing foods, thus acting as a reminder and has the potential to have a high impact on the control of food-borne helminthiases. The *Rhyme of Poyang Lake* video (first class in the audiovisual category) imparts knowledge of schistosomiasis through a story. It has the potential to have a strong impact, as it urges the acceptance of knowledge and to make behavioral changes through the demonstration of the harm. The first-class product in the comprehensive category on the prevention of echinococcosis integrates diverse products and takes into consideration the characteristics of the minority (e.g. through the application of language and selection of household objects).

This survey had several limitations. First, some products have already been piloted in endemic areas, while others have not yet been assessed scientifically. Thus, the evaluation of the cost-effectiveness indicator was largely based on the experts’ specialty and experiences, which suggests that it is necessary to evaluate the effectiveness of these health education products through evidence-base pilots [15]. The pilots could lead to an improvement of the products, which will benefit wide distribution. Secondly, the population size accessing to the products is important to evaluate the products. However, this could not be provided by most products. On the one hand, many products are designed by provincial-level bodies, which are then reproduced by different counties. On the other hand, audiovisual products are usually broadcast through public platform, e.g. television. Thus, it is challenging to estimate what portion of the population is covered by many of the products. Thirdly, most products focus on schistosomiasis and echinococcosis, while there is an inadequate number of products aimed at other helminthiases. For example, clonorchiasis is now the most important food-borne helminthiasis in China, causing a huge disease burden in endemic areas [9, 10]. Notably no single product focusing on clonorchiasis was awarded, which shows a lack of high quality products focusing on this disease. Thus, experiences from the schistosomiasis and echinococcosis products should be drawn upon to design products for clonorchiasis as well as other helminthiases.

## Conclusions

Diverse health education products have been designed and applied in the control of helminthiases in China, however, most belong to the graphic design and daily-use categories. Provincial- and county-level CDCs and IPDs are the major designers and makers. Most products were found to be on schistosomiasis and echinococcosis, which indicates the priority of these diseases in China. Most products took into consideration the characteristics of targeted disease(s) and population, a good feature showing specificity.

There are significant gaps, however, in terms of both the quantity and quality of products for some diseases. The experiences from several first-class awarded products could be drawn upon to design products for those diseases without adequate health education products, e.g. clonorchiasis. The suggestions for education products aimed at controlling and preventing helminthiases are listed in Table 3. Additionally, this study is also expected to promote the control of helminthiases globally. Similar surveys could be advocated for in other countries. In addition, the awarded products deserve to be adapted to and tested in other endemic countries.

**Table 3:** Suggestions for health education products aimed at controlling and preventing helminthiases

1. *Attention to pre-design and pre-application:*	
Before products are designed, surveys should be implemented to capture the qualitative and quantitative information related to knowledge, attitudes, practices, beliefs, culture, and psychology of different populations. Before wide distribution, education products should be piloted.	
2. *The combination of style and content*:	
The style determines the attractiveness and acceptability. Only after being attracted, people then have the will to access the content. The content determines whether knowledge can be distributed scientifically and intelligibly, and whether the guides on behavior change are acceptable.	
3. *Integration of multiple products*:	
Due to the differences among and between populations (including in terms of knowledge, culture, and psychology), multiple products are recommended to target different populations, even for a single disease.	
4. *Attention to universality and specificity:*	
Products with high specificity could increase acceptability in targeted areas and populations. Universality allows for products to be suitable for diverse situations, which reduces challenges and costs related to design and production. The balance on universality and specificity should be strived for.	
5. *Consideration of distribution platforms:*	
The platforms for future product distribution should be taken into consideration during the design stage, which would impact acceptability and subsequent coverage.	

## Additional files

Additional file 1: Multilingual abstracts in the five official working languages of the United Nations. (PDF 949 kb)

Additional file 2: **Table S1.** Evaluation indicators and corresponding weights of graphic design products. **Table S2.** Evaluation indicators and corresponding weights of daily-use products. **Table S3.** Evaluation indicators and corresponding weights of audiovisual products. **Table S4.** The first round screening of all products (DOCX 25 kb)

## Abbreviations

CDCs: Centers for Disease Control and Prevention
IPDs: Institutes or control stations of parasitic diseases
