# Supplementary material for: Assessment of health education products aimed at controlling and preventing helminthiases in China
Source: Infect Dis Poverty. 2019 Mar 26;8:22. doi: 10.1186/s40249-019-0531-y (PMC6434872; doi:10.1186/s40249-019-0531-y)
Supplement: Supplementary file 1 — Multilingual abstracts in the five official working languages of the United Nations. (PDF 949 kb) [file 40249_2019_531_MOESM1_ESM.pdf]

Translation of the abstract into the five official working languages of the United Nations

ويهدف تقييم منتجات التثقيف الصحي إلى مكافحة داء الديدان الطفيلية والوقاية منها في الصين

من باو تشيان، تشانغ هاي تشو، هوي هوي تشو، تينغ يون تشو، جي لي هوانغ، ينغ دان تشن، شياو نونغ تشو

ملخص

معلومات أساسية: ألقى داء الديدان الطفيلية عبئًا ثقيلًا على كاهل السكان في الصين. ومع ذلك، أدى انتشار أنشطة المكافحة الى إحراز إنجازات هامة. وبما أن جهود التثقيف الصحي قد لاقت انتشارًا واسعًا وتؤدي دورًا مهمًا في مكافحة هذه الأمراض وإزالة منها، عكفنا على جمع منتجات التثقيف الصحي التي تهدف إلى مكافحة داء الديدان الطفيلية والوقاية منها في الصين. وقمنا بتحليل خصائصها وتقييم جودتها.

الأساليب: أولًا، قمنا بجمع منتجات التثقيف الصحي التي تهدف إلى مكافحة داء الديدان الطفيلية والوقاية منها من مجموعة متنوعة من المنظمات. ثانيًا، طبقنا أسلوبي تقارع الأفكار بين الخبراء وأساليب دلهي لوضع نظام تقييم، الذي استخدام بعد ذلك لتقييم المنتجات التي جمعت تقييمًا منهجيًا. ومنحت المنتجات التي ثبُت أنها ممتازة. الخصائص – وتشمل نوع المنتجات التي جمعت ومصدرها والمرض/الأمراض المستهدفة والسكان المستهدفين ولغاتهم – من جمع منتجات وتعرض هنا أيضًا المنتجات الممنوحة.

النتائج: جُمِع 96 منتج تثقيف صحي إجمالًا عن داء الديدان الطفيلية من 53 منظمة. واندرج معظم هذه المنتجات إما ضمن فئة التصميم الشكلي (47) أو الاستخدام اليومي (24). جُمع سبعون منتجًا منها من مراكز مكافحة الأمراض والوقاية منها و20 منتجًا من المعاهد أو مراكز مكافحة الأمراض الطفيلية، في الأساس على المستوى المقاطعات والبلديات. فيما يتعلق بالأمراض التي تستهدفها المنتجات ، ركز 67 منتجًا على الديدان الطفيلية الفردية، و25 منتجًا على الديدان الطفيلية المتعددة، وركز الأربعة المتبقية على أمراض غير محددة. من 67 منتجًا التي تركز على داء الديدان الطفيلية فقط، استهدف معظمها البلهارسيا (37)، يليها داء المشوكات (16). استهدفت غالبية المنتجات (79) عامة السكان، بينما استهدف 11 منتجًا الطلاب على وجه الخصوص. فيما يتعلق باللغات، كان 86 منتجًا باللغة الصينية فقط، في حين أن المنتجات العشرة الأخرى كانت باللغتين الصينية ولغات الأقليات في الصين. من هذه المنتجات العشرة، استهدف أحدها البلهارسيا واستهدف التسعة الأخرى داء المشوكات. مُنح أربعة وثلاثون منتجًا منها. وكانت خصائص المنتجات الممنوحة مماثلة لخصائص المنتجات التي جمعت.

الاستنتاجات: صممت مجموعة متنوعة من منتجات التثقيف الصحي وطبقت للوقاية من الديدان الطفيلية ومكافحتها في الصين. العديد من هذه المنتجات لها ميزات جيدة مثل تحديد الأمراض والسكان المستهدفين. ومع ذلك، هناك فجوات كبيرة من حيث كمية ونوعية المنتجات المتعلقة ببعض الأمراض. ويمكن الاستفادة من تجارب المنتجات الممنوحة لتصميم المزيد من المنتجات التي تستهدف مجموعة من أنواع الديدان المختلفة.

Translated from English version into Arabic by Saad Mohamed, proofread by Aaminah Natha, through

TRANSLATORS WITHOUT BORDERS

中国用于蠕虫病控制的健康教育产品的评价

钱门宝，周长海，朱慧慧，诸廷俊，黄继磊，陈颖丹，周晓农

摘要

引言：蠕虫病曾经在中国造成显著的疾病负担。但是由于广泛的控制措施，其防控取得了显著的成就。健康教育得到了大范围的传播并且在蠕虫病控制和消除过程中发挥了重要的作用。我们收集了中国用于蠕虫病防控的健康教育产品、分析了其特征并且评价了其质量。

方法：首先，从多个机构收集用于蠕虫病防控的健康教育产品。其后，通过专家头脑风暴法和德尔菲法建立了质量评价体系，对收集的产品进行了系统的评价，并对优秀产品进行了奖励。本文分析了收集的产品以及获奖产品的特征，包括类型、来源、目标病种、目标人群和语言。

结果：从 53 个机构收集了 96 组产品。大部分产品属于平面类（47 组）和实物类（24 组）。70 组产品由疾病预防控制中心提交，20 组作品由寄生虫病防治所（防治站）提交，且主要来自省级和县级。67 组产品针对单一寄生虫病，25 组产品针对多种寄生虫病，剩余 4 组未针对特定病种。67 组针对单一寄生虫病的产品中，37 组针对血吸虫病，16 组针对棘球蚴病。79 组产品针对普通人群，11 组产品针对学生人群。86 组产品使用汉语，剩余 10 组产品同时使用汉语和少数民族语言。这 10 组产品中，1 种针对血吸虫病，其他 9 种针对棘球蚴病。34 组产品获奖，这些获奖作品的特征与总体收集到的产品特征类似。

结论：为预防和控制蠕虫病，在中国设计和应用了多样的健康教育产品。其中很多产品具有优秀的特征如针对特定病种和人群。但是，对于某些寄生虫病，其健康教育产品的数量和质量均

显不足。需要从那些获奖作品中提炼经验，从而为其他寄生虫病设计更多的健康教育产品。

Translated from English version into Chinese by Men-Bao Qian

Évaluation de matériel d'éducation sanitaire sur la lutte contre les helminthiases et leur prévention en Chine

Men-Bao Qian, Chang-Hai Zhou, Hui-Hui Zhu, Ting-Jun Zhu, Ji-Lei Huang, Ying-Dan Chen, Xiao-Nong Zhou

Résumé

Contexte: Les helminthiases font peser un fardeau de morbidité énorme sur la population chinoise, mais les activités de lutte généralisées ont produit des résultats significatifs. Parce que l'éducation sanitaire, largement diffusée, a joué un rôle important dans la lutte contre ces maladies et leur élimination, nous avons collecté du matériel d'éducation sanitaire consacré à la lutte contre les helminthiases et à leur prévention en Chine. Nous avons analysé leurs caractéristiques et évalué leur qualité.
Méthodes: Dans un premier temps, du matériel d'éducation sanitaire concernant la lutte contre les helminthiases et leur prévention ont été collectés auprès d'organismes très divers. Dans un deuxième temps, nous avons employé des groupes de réflexion d'experts et la méthode de Delphes pour élaborer un système d'évaluation, qui a ensuite été utilisé pour évaluer systématiquement le matériel d'éducation collecté. Les supports éducatifs jugés excellents ont été récompensés. Les caractéristiques (type, source, maladie(s) ciblée(s), population visée, langues, etc.) du matériel collecté et des produits récompensés sont présentées ici.
Résultats: Au total, 96 supports d'éducation sanitaire concernant les helminthiases ont été recueillis auprès de 53 organismes. La plupart des produits appartenaient à la catégorie « création graphique » (47) ou « utilisation quotidienne » (24). Soixante-dix produits ont été collectés auprès des Centres de lutte et de prévention des maladies et 20 auprès d'instituts ou de postes de lutte contre les maladies parasitaires, principalement au niveau des provinces et des circonscriptions. En ce qui concerne les maladies abordées dans ces documents, 67 concernaient une seule forme d'helminthiase, 25 plusieurs formes et les 4 autres des maladies non précisées. Parmi les 67 supports concernant les helminthiases, la schistosomiase était la plus souvent visée (37), suivie de l'échinococcose (16). La majorité des supports (79) s'adressaient à la population générale, mais 11 visaient spécifiquement les écoliers. En ce qui concerne les langues, 86 supports étaient uniquement en chinois, tandis que les 10 autres étaient à la fois en chinois et dans des langues minoritaires de la Chine. Sur ces dix derniers supports, l'un concernait la schistosomiase et les neuf autres, l'échinococcose. Trente-quatre produits ont été attribués. Leurs caractéristiques étaient similaires à celles du matériel collecté dans son ensemble.
Conclusions: Des supports d'éducation sanitaire très divers ont été conçus et employés pour la prévention et la lutte contre les helminthiases en Chine. Ce matériel présente souvent de bonnes caractéristiques, par exemple l'indication de la maladie visée et des populations ciblées. Cependant, le nombre et la qualité des supports concernant certaines maladies sont très insuffisants. Le retour d'expérience sur les supports éducatifs récompensés pourrait être mis à profit pour concevoir davantage de matériel concernant plusieurs helminthiases différentes.

Translated from English version into French by Iris Soliman, proofread by Suzanne Assenat, through

TRANSLATORS
WITHOUT BORDERS

Оценка изделий для санитарного просвещения, направленных на борьбу и предупреждение гельминтоза в Китае

Men-Bao Qian (Мен-Бао Цянь), Chang-Hai Zhou (Чан-Хай Чжоу), Hui-Hui Zhu (Хуэй-Хуэй Чжу), Ting-Jun Zhu (Тин-Цзюнь Чжу), Ji-Lei Huang (Джи-лей Хуан), Ying-Dan Chen (Ин-Дан Чен), Xiao-Nong Zhou (Сяо-Нонг Чжоу)

Аннотация

Справочная информация: гельминтоз достиг огромного уровня заболеваемости среди китайского населения. Однако, широкие меры по борьбе с ним привели к значительным

достижениям. По мере распространения санитарного просвещения и его ключевой роли в борьбе и устранении этих заболеваний, мы собрали изделия для санитарного просвещения, которые направлены на борьбу и предотвращение гельминтоза в Китае. Мы проанализировали их характеристики и оценили их качество.
Методы: Во-первых, мы собрали от различных организаций изделия для санитарного просвещения, направленные на борьбу с гельминтозом и его предупреждение. Во-вторых, был применен метод экспертного обсуждения и метод Делфи для разработки системы оценки, которая в последствии использовалась для систематической оценки собранных изделий. Наград удостоились изделия с оценкой отлично. Здесь представлены характеристики полученных и награжденных изделий, включая тип, происхождение, целевое(ые) заболевание(я), целевое население и языки.
Результаты: всего от 53 организаций было собрано 96 изделий для санитарного просвещения о гельминтозе. Большинство изделий относились к графическому дизайну (47) или к категории ежедневного употребления. 70 изделий были предоставлены Центром по борьбе и предупреждению заболеваний, а 20 — институтами или пунктами профилактики паразитарных заболеваний, в основном на уровне провинций и округов. Что касается заболеваний, на борьбу с которыми направлены эти изделия, то 67 направлены на борьбу с одиночным гельминтозом, 25 — с множественным гельминтозом, а остальные 4 — с неспецифическими заболеваниями. Из 67 изделий, направленных на борьбу с одиночным гельминтозом, большая часть (37) нацелена на борьбу с шистосомозом, а остальные (16) — с эхинококкозом. Большинство изделий (79) были нацелены на население в целом, а 11 — конкретно на студентов. Относительно языков, 86 изделий были на китайском языке, а оставшиеся 10 — как на китайском так и на языках национальных меньшинств Китая. Из этих 10 изделий 1 было нацелено на борьбу с шистосомозом, а остальные 9 — с эхинококкозом. Наградами были удостоены 34 изделия. По характеристикам, награжденные изделия были схожи с изделиями, полученными от организаций.
Заключение: было создано и применено множество изделий для санитарного просвещения, направленных на борьбу и предупреждение гельминтоза в Китае. Многие изделия отличаются такими полезными характеристиками, как определение целевых заболеваний и групп населения. Однако существуют значительные недостатки с точки зрения количества и качества изделий, имеющих отношение к некоторым из этих заболеваний. Можно позаимствовать опыт создания изделий, получивших награды, для разработки большего числа изделий, направленных на борьбу с различными видами гельминтоза.

Translated from English version into Russian by Gunel Huseynbayova, proofread by Michael Orlov, through

TRANSLATORS
WITHOUT BORDERS

Evaluación de productos de educación sanitaria destinados a controlar y prevenir las helmintiasis en China

Men-Bao Qian, Chang-Hai Zhou, Hui-Hui Zhu, Ting-Jun Zhu, Ji-Lei Huang, Ying-Dan Chen, Xiao-Nong Zhou

Resumen
Antecedentes: Las helmintiasis han impuesto un enorme peso en la salud de la población de China. Sin embargo, las actividades generalizadas de control han tenido como resultado logros significativos. Como la educación sanitaria ha tenido una amplia difusión y desempeña un papel importante en el control y la eliminación de estas enfermedades, recopilamos productos de educación para la salud destinados a controlar y prevenir las helmintiasis en China. Analizamos sus características y evaluamos su calidad.
Métodos: En primer lugar, los productos de educación para la salud destinados a controlar y prevenir las helmintiasis se obtuvieron de una amplia gama de organizaciones. En segundo lugar, se aplicaron los métodos tormenta de ideas y Delfos para establecer un sistema de evaluación, que luego se utilizó para evaluar los productos recolectados de manera sistemática. Aquellos considerados excelentes fueron premiados. Se presentaron aquí las características de los productos recolectados, incluidos el tipo, la fuente, la(s) enfermedad(es) objetivo, la población objetivo y los idiomas, y los productos premiados.
Resultados: En total, se recolectaron 96 productos de educación sanitaria sobre la helmintiasis de 53 organizaciones. La mayoría de los productos pertenecían a las categorías dediseño gráfico (47) o de uso

diario (24). Setenta fueron recolectados de los Centros para el Control y Prevención de Enfermedades y 20, de institutos o estaciones de control de enfermedades parasitarias, principalmente a nivel provincial y de condado. Con respecto a las enfermedades objetivo de los productos, 67 se centraron en una helmintiasis simple, 25 en helmintiasis múltiples y las cuatro restantes, en enfermedades no específicas. De los 67 productos centrados en la helmintiasis simple, la mayoría tuvo como objetivo la esquistosomiasis (37), seguida por la equinococosis (16). La mayoría de los productos (79) se dirigían a la población general, mientras que 11 se enfocaban específicamente en los estudiantes. En cuanto a los idiomas, 86 productos estaban solo en chino, mientras que los otros diez estaban tanto en chino como en los idiomas minoritarios de China. De estos diez productos, uno tenía como objetivo la esquistosomiasis y los otros nueve a la equinococosis. Treinta y cuatro productos fueron premiados. Las características de los productos premiados eran similares a las de los productos recolectados.

Conclusiones: Se ha diseñado y aplicado una amplia gama de productos de educación para la salud para la prevención y el control de las helmintiasis en China. Muchos productos tienen buenas características, tales como aclarar a cuáles enfermedades y poblaciones específicas están dirigidos. Sin embargo, existen brechas significativas en cuanto a la cantidad y calidad de los productos respecto con algunas de las enfermedades. La experiencia con los productos premiados podría aprovecharse para diseñar más productos dirigidos a diversas helmintiasis.

Translated from English version into Spanish by Juan Aquino, proofread by Maria Paula Gorgone, through

TRANSLATORS WITHOUT BORDERS
