# Supplementary material for: Assessment of health education products aimed at controlling and preventing helminthiases in China
Source: Infect Dis Poverty. 2019 Mar 26;8:22. doi: 10.1186/s40249-019-0531-y (PMC6434872; doi:10.1186/s40249-019-0531-y)
Supplement: Supplementary file 2 — Table S1. Evaluation indicators and corresponding weights of graphic design products. Table S2. Evaluation indicators and corresponding weights of daily-use products. Table S3. Evaluation indicators and corresponding weights of audiovisual products. Table S4. The first round screening of all products (DOCX 25 kb) [file 40249_2019_531_MOESM2_ESM.docx]

**Additional file 2:**

***Table S1:*** Evaluation indicators and corresponding weights of graphic design products

| **Primary indicators** | | **Secondary indicators** | |
| --- | --- | --- | --- |
| **Indicators** | **Weight** | **Indicators** | **Weight** |
| 1. Scientificity | 4.45 | 1.1 Accuracy of information | 4.42 |
|  |  | 1.2 Guidance for behavior | 4.41 |
|  |  | 1.3 Key points | 4.29 |
| 2. Creativity | 3.75 | 2.1 Layout | 3.61 |
|  |  | 2.2 Theme | 4.17 |
|  |  | 2.3 Colour | 3.02 |
| 3. Acceptability | 4.25 | 3.1 Easy to understand | 4.44 |
|  |  | 3.2 Combination of text and pictures | 4.56 |
|  |  | 3.3 Feasibility | 4.07 |
| 4. Generalizability | 4.06 | 4.1 Cost-effectiveness | 3.40 |
|  |  | 4.2 Applicability in different populations and areas | 3.54 |
|  |  | 4.3 Application situation | 3.19 |

***Table S2:*** Evaluation indicators and corresponding weights of daily-use products

| **Primary indicators** | | **Secondary indicators** | |
| --- | --- | --- | --- |
| **Indicators** | **Weight** | **Indicators** | **Weight** |
| 1. Scientificity | 4.28 | 1.1 Accuracy of information | 4.20 |
|  |  | 1.2 Guidance for behavior | 4.13 |
|  |  | 1.3 Pertinence | 4.04 |
| 2. Creativity | 3.42 | 2.1 Fashion and attractiveness | 3.26 |
|  |  | 2.2 Environment-friendly materials | 3.23 |
|  |  | 2.3 Delicate design | 3.66 |
| 3. Acceptability | 4.42 | 3.1 Practicability | 4.21 |
|  |  | 3.2 Convenience | 3.89 |
|  |  | 3.3 Popularity | 4.09 |
| 4. Generalizability | 3.83 | 4.1 Cost-effectiveness | 3.68 |
|  |  | 4.2 Applicability in different populations and areas | 3.77 |
|  |  | 4.3 Application situation | 3.37 |

***Table S3:*** Evaluation indicators and corresponding weights of audiovisual products

| **Primary indicators** | | **Secondary indicators** | |
| --- | --- | --- | --- |
| **Indicators** | **Weight** | **Indicators** | **Weight** |
| 1. Scientificity | 4.33 | 1.1 Accuracy of information | 4.37 |
|  |  | 1.2 Guidance for behavior | 4.25 |
|  |  | 1.3 Pertinence | 4.18 |
| 2. Creativity | 3.73 | 2.1 Attractive title | 3.68 |
|  |  | 2.2 Being episodic in narrative | 3.80 |
|  |  | 2.3 Vivid pictures | 3.88 |
| 3. Acceptability | 4.19 | 3.1 Interesting | 3.98 |
|  |  | 3.2 Proper emotional punch | 3.59 |
|  |  | 3.3 Proper duration | 3.70 |
| 4. Generalizability | 4.00 | 4.1 Cost-effectiveness | 3.37 |
|  |  | 4.2 Applicability in different populations and areas | 3.62 |
|  |  | 4.3 Application situation | 3.08 |

***Table S4:*** The first round screening of all products

| **Types** | **Number of collected products** | **Suggested number for second-round evaluation** | **Final number for second-round evaluation^a^** |
| --- | --- | --- | --- |
| Graphic design products | 47 | 15 | 19 |
| Daily-use products | 24 | 10 | 11 |
| Audio-visual products | 11 | 8 | 8 |
| Comprehensive products | 14 | 8 | 8 |

a: There existed equal votes, and those with the equal votes were all included in the second round.
